# Barriers to a cure for HIV in women

**DOI:** 10.7448/IAS.19.1.20706

**Published:** 2016-02-18

**Authors:** Sara Gianella, Athe Tsibris, Liz Barr, Catherine Godfrey

**Affiliations:** 1 Division of Infectious Diseases, School of Medicine, University of California San Diego, La Jolla, CA, USA; 2 Division of Infectious Diseases, Brigham and Women's Hospital, Harvard Medical School, Boston, MA, USA; 3 AIDS Clinical Trials Group Community Scientific Subcommittee, Madison, Wisconsin, USA; 4 Division of AIDS, National Institutes of Health, Bethesda, MD, USA

**Keywords:** HIV eradication, HIV cure, women's health, immune system, female genital tract, anatomic compartments

## Abstract

**Introduction:**

Distinct biological factors exist that affect the natural history of HIV and the host immune response between women and men. These differences must be addressed to permit the optimal design of effective HIV eradication strategies for much of the HIV-positive population.

**Methods and results:**

Here, we review the literature on sex-based differences in HIV pathogenesis and natural history in tissues and anatomic compartments, HIV latency and transcriptional activity, and host immunity including the role of sex hormones. We then outline the potential effects of these differences on HIV persistence, and on the safety and efficacy of HIV eradication and curative interventions. Finally, we discuss the next steps necessary to elucidate these factors to achieve a cure for HIV, taking in account the complex ethical issues and the regulatory landscape in the hopes of stimulating further research and awareness in these areas.

**Conclusions:**

Targeted enrolment of women in clinical trials and careful sex-based analysis will be crucial to gain further insights into sex-based differences in HIV persistence and to design sex-specific approaches to HIV eradication, if required.

## Introduction

Antiretroviral therapy (ART) suppresses virus replication in HIV-positive individuals and reduces morbidity and mortality [1]. ART cannot eliminate cells harbouring HIV DNA [2] and plasma viremia, in general, rebounds quickly after treatment is interrupted [3]. Replication-competent HIV DNA that remains integrated in long-lived cells despite effective ART, referred to as the HIV reservoir, represents the major barrier to an HIV cure [4]. Two paradigms are currently under evaluation for HIV cure: 1) reactivation of HIV transcription from latently infected cells followed by a second step that leverages pharmacological agents, genetic engineering, and/or immunological approaches to clear the reservoir and 2) permanent silencing of latently HIV-infected cells [5]. Differences in sex, age, and race may influence the HIV reservoir and the host immune response to these therapeutic approaches as a consequence of hormone fluctuations, anatomic characteristics, genetic differences, drug responses, and other factors [6,7]. Such factors could also affect the safety and efficacy of curative interventions. Here, we review the literature on the fundamental differences between men and women in the immunologic response to HIV infection and pathogenesis, and we discuss the next steps necessary to elucidate these factors in order to achieve HIV eradication.

## Discussion

### Epidemiology of HIV in women

Globally, women account for nearly half of all people living with HIV and represent even a greater proportion of those affected in low- and middle-income countries [8]. Gender inequalities and harmful gender norms that promote unsafe sex and limit access to health services, education, and economic opportunities continue to drive the HIV epidemic in many countries [9]. As a consequence, the prevalence of HIV among girls and young women is more than double that of similarly aged males [10,11]. Despite this, women are still largely underrepresented in HIV cure research. Most cure-related research and clinical trials take place in developed countries, where the HIV epidemic is predominantly driven by men who have sex with men [12,13]. Therefore, differences in barriers to eradicating HIV between men and women have not been adequately considered. More data are needed to evaluate these potential differences and how they may affect the safety and efficacy of curative interventions.

### Sex differences in the pathogenesis and natural history of HIV infection

Sex differences exist in biomarkers for HIV infection and disease progression. Among HIV-negative women, the absolute count and percentage of CD4^+^ T cells are significantly higher than in heterosexual men, and fluctuations in percentage of CD4^+^ T cells have been described with the menstrual cycle [14]. Similarly, HIV-positive women have higher CD4^+^ T cell counts [15–17] and lower plasma HIV RNA levels [15,18–20] when compared to men during both the acute and chronic phases of HIV infection. A greater proportion of women in HIV controller cohorts has been described [21,22]. Nevertheless, women have similar rates of progression to AIDS as men [18,19]. Although socio-economic differences may be critical in HIV acquisition and disease progression [7], there is a growing body of information identifying key biological factors which are likely to be partially responsible for the observed sex-based differences. For example, women report or experience fewer symptoms during primary infection, which may cause a delay in diagnosis of HIV in this population [15]. Differences in immunologic responses to HIV infection, mediated through sex hormones and genetic variation, have also been implicated as possible mechanisms [7]. Women more frequently report ART side effects and ART discontinuation compared to HIV-positive males [23–32], and this may be important in accelerating HIV disease progression. Despite known sex-specific differences in the natural history of HIV disease, the impact of these factors on the establishment and maintenance of the HIV reservoir and how these differences may affect HIV eradication strategies are poorly understood. Table 1 summarizes the current knowledge gaps regarding sex-based differences in the HIV reservoir that warrant further investigation.

**Table 1:** Current knowledge gaps regarding sex-based differences in the HIV reservoir

Establishment and maintenance of HIV latency
Reservoir size and dynamics
HIV reservoir distribution across cells and anatomic compartments
Host immune response
Impact of adolescence and menopause on the HIV reservoir
Impact of pregnancy and lactation on the HIV reservoir
Natural hormone effects on HIV transcriptional reactivation
Exogenous hormone therapy effects on the HIV reservoir
Sex-based differences in eradication strategies

### Sex differences in the immune pathogenesis

Sex differences in the immune response are related to both the differential expression of regulatory genes on the X- and Y-chromosomes and the direct effect of sex hormones on target cells [33–35]. Key regulatory genes involved in the immune response are located on the X-chromosome, and X-chromosome inactivation has evolved as a mechanism to equalize gene expression dosage between males and females [33]. Elevated levels of functional immune proteins in women can result when X-chromosome genes involved in immune response escape silencing [33,36,37].

**Figure 1:**
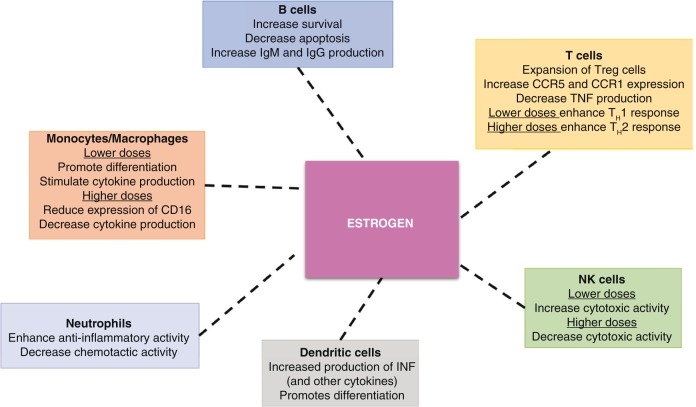
Effect of oestrogen on immune cells, as T cells, B cells, dendritic cells, Monocytes/Macrophages, neutrophils and natural killer (NK) cells.

Sex hormones also play an important role in modulating the immune response. The role of oestrogens in the adaptive immune response has been intensively studied in the context of autoimmune diseases, where women are at significantly greater risk for disease (Figure 1) [38]. Sex differences have also been investigated in the setting of vaccine administration, with women generally having a more robust immune response, higher titer of antibodies and enhanced interferon (IFN) response when compared to men [39].

There appears to be a positive dose response relationship between higher levels of oestrogens and circulating immunoglobulins [40]. This effect is mediated through oestrogenic stimulation of IL-10 production by monocytes and subsequent B-cell stimulation [40] and differentiation [41,42]. Studies suggest that oestrogens up-regulate the expression of mediators of B-cell survival while reducing mediators of B-cell apoptosis, such as PD-1 [43,44]. Oestrogens also modulate other immune cells, especially T lymphocytes and monocytes. Physiologic levels of oestrogens stimulate the expansion of CD4^+^ regulatory T cells, especially during the ovarian follicular phase [45] and these cells play an important role in maintaining immune homeostasis during viral infections [46–48]. Oestrogens also exert a dose-dependent effect on T helper 1 (TH1)-cell and TH2-cell differentiation. Specifically, low doses of oestrogens are associated with TH1-cell responses and enhanced cell-mediated immunity, whereas high doses of oestrogens promote TH2-cell and humoral responses [33]. Interestingly, there is evidence to suggest that the contrasting effects of oestrogens on T-bet (a master regulator of TH1-cell differentiation), and IFN regulatory factor-1 (a transcription factor that is associated with the regulation of IFN-γ), may be dose-dependent and account for TH1-cell versus TH2-cell differentiation [33,49]. Higher doses of oestrogens selectively increase the expression of CC-chemokine receptor 5 (CCR5) and CCR1 on CD4^+^ T cells, while decreasing tumour-necrosis factor (TNF) production [33]. There also seems to be a biphasic effect of oestrogen on monocyte activity: lower concentrations can enhance the production of pro-inflammatory cytokines, whereas higher concentrations are associated with reduced expression of CD16 and less production of the pro-inflammatory cytokines interleukin (IL)-1β, IL-6, and TNF [50,51]. Similarly, natural killer (NK) cells demonstrate increased cytotoxic activity in the presence of physiologic doses of oestrogens, but decreased cytotoxicity when oestrogen levels are higher. Finally, oestrogens seem to exert an anti-inflammatory effect on neutrophils and a suppressive effect on their chemotactic activity [33]. These observations may account for the excess morbidity associated with certain viral infections during pregnancy [52,53]

Less is known about the effect of progestins in this context [54]. The general function of progesterone is to preserve pregnancy and, in pregnancy, it exerts an immune suppressive and anti-inflammatory function [54]. However, the relationship between progestins and the immune system is complex [54]. Progesterone receptors are found on various immune cells (e.g. T cells, macrophages, and plasmacytoid dendritic cells) [54]. During the luteal phase of the menstrual cycle, progesterone receptors are increased on CD8^+^ T cells [55] and progestins may directly inhibit NK cell function inducing apoptosis [56]. During the secretory phase, progestins are associated with an increase in uterine NK cells, macrophages, and mature dendritic cells [56]. Synthetic progestins such as those used in contraceptive agents can up-regulate several genes involved in microbial recognition and antiviral response significantly more than natural progesterone [57]. A recent study found that injectable progestin-only contraceptives and high endogenous progesterone were both associated with increased frequency of activated HIV targets cells in the cervix, providing a possible mechanism to explain increased HIV acquisition [58]. The effects of progestins on HIV reservoir size and dynamics are unknown. A better understanding of the intricate immunomodulatory effects of sex hormones (oestrogens and progestin) and their relevance to an eradication strategy is warranted.

Several *in vitro* and *in vivo* studies reported higher levels of Toll-like receptor 7-mediated IFN-α production from plasmacytoid dendritic cells in HIV-positive women compared to men [59,60], likely as a consequence of stronger induction of IFN-stimulated genes [61]. As a result, women have greater levels of activated CD8^+^ T cells than men for a given HIV viral load [7]. HIV-positive women typically have higher levels of D-dimers [62], more pronounced immune responses after vaccine administration [60,61,63], and higher levels of several markers of innate immune activation compared to men [64–66]. Sex-based differences in the inflammatory response might explain the observed differences in the clinical manifestations of HIV infection, including better control of the HIV viremia during primary infection and accelerated disease progression during chronic infection [7]. Because increased immune activation is also associated with a larger HIV reservoir [67,68], the increased immune activation experienced by women may be important when considering eradication strategies. Chronic inflammation may also promote clonal expansion of HIV-infected cells [67]. Taken together, these observations suggest that there are competing effects of chronic inflammation and the viral immune response in women and these sex differences need to be considered when designing eradication strategies [33].

### Sex differences in HIV reservoirs in tissues and anatomic compartments

Current evidence suggests that the HIV DNA burden is not uniformly distributed within the human body. HIV DNA levels are approximately four-fold higher in the gut, relative to blood [69]. These levels vary across gut sites (terminal ileum, colon, duodenum, and rectum) but uniformly exceed levels in peripheral blood mononuclear cells (PBMC) [70–72]. Regarding lymph nodes, data suggest that the HIV reservoir burden is similar to or exceeds that found in blood [73–76]. Persistent HIV replication has been detected in lymph nodes (and other anatomic reservoirs) despite ART [77,78], suggesting that HIV reservoirs in these locations may remain transcriptionally active even when HIV RNA is undetectable in plasma. Because of the marked physiologic differences between sexes (hormonal, metabolic, fat distribution, immunologic, and pharmacokinetic) and recent data on the effects of oestrogen on HIV transcriptional activation (discussed in section 8 below) it is conceivable that the location and the amounts of replication-competent HIV may be different in men and women and that these differences may be relevant to future curative efforts. For example, the relationship between the amounts of replication-competent HIV in blood and in the female reproductive tract is unknown (see also section 6 below) and sex differences in the HIV reservoir distribution between gut, lymph nodes, and other tissues are currently under investigation. One recent study found a high proportion of activated CD4^+^ T cells harbouring HIV DNA in adipose tissue, suggesting that this might be an additional reservoir to be considered [79]. Men and women have well-documented differences in fat content and adipocyte function is modulated by oestrogens [80,81]. Another potentially important HIV reservoir is the central nervous system (CNS). All steroids, including sex hormones, affect several critical properties of the blood brain barrier, including cellular efflux mechanisms, nutrient uptake, and tight junction integrity. Such actions not only influence brain homeostasis but also the delivery of CNS-targeted therapeutics and cellular migration, and perhaps also the size and distribution of the HIV reservoir within the CNS [82].

### The female genital tract

The female genital tract is a complex immunological and microbial milieu comprising separate anatomic compartments for the upper and lower genital tract with different environments [83]. There is no equivalent of these compartments for men and both the upper and lower genital tract have features that allow for pregnancy, change the risk environment for HIV acquisition, and may be important when considering the size and nature of the HIV reservoir in women. Although the presence of ongoing viral replication in blood or gastrointestinal tissue during suppressive ART remains controversial [84–88], the evidence for virus production in the female genital tract when HIV RNA levels are undetectable in blood plasma has been described, especially in the setting of local inflammation [89,90]. In the lower female genital tract, the microbiome plays an important role in determining the risk of acquisition and transmission of a variety of diseases including sexually transmitted infections and ascending infections of the upper genital tract [91]. Colonization with hydrogen peroxide-producing *Lactobacillus* maintains vaginal health and is associated with a reduced inflammatory environment [92], whereas bacterial microenvironments with a great deal of species diversity (as in bacterial vaginosis) are associated with increased production of inflammatory cytokines [93], greater numbers of activated immune cells [94], and enhanced HIV RNA shedding [93,95–97].

Similarly, clinically relevant viruses such as herpesviruses and human papillomavirus (HPV) are also associated with increased vaginal inflammation and immune activation. Although both men and women are susceptible to these viruses, their interaction with the vaginal microbiome may specifically affect the risk environment in women. For example, HPV acquisition and persistence is associated with greater levels of inflammatory cytokines [98], and there is growing evidence associating HPV infection with an increased risk of HIV acquisition [98,99], presumably related to the genital recruitment of immune cells, which appears to be independent from other sexually transmitted infections and risk behaviour [100,101]. The exact interplay between sex hormones, mucosal immunity, and the resident microbial flora in the female reproductive tract and their effect on the HIV reservoir remains inadequately understood and requires further investigation. Specific factors associated with increased genital inflammation and recruitment of (activated) immune cells are likely to be different in men and women. These immune cells serve as potential targets for HIV and likely play an important role in the maintenance of the genital HIV reservoir in both sexes.

Immune cell distribution in the female reproductive tract has been recently reviewed [102] and significant differences exist between the uterine/endocervix and the vagina/ectocervix environment, when compared to peripheral blood. T cell populations in the genital tract consist of a relative increase in CD8^+^ T cells (relative to CD4^+^ T cells) and more macrophages compared to peripheral blood. Increased antigen presenting cells and activated CCR5^+^CD4^+^ T cells in the cervix have been described in the setting of high diversity vaginal microbiota [93]. Vaginal macrophages express more CD4 receptors and have an increased density of CCR5 and CXCR4 co-receptors compared to those residing in the gut [103], which represents a significant enrichment of activated target cells in the vaginal milieu. The extent to which these cells harbour replication-competent HIV is unknown and requires further study.

The immune milieu of the uterus has been studied in healthy women and the proportion of activated CD4^+^ T cells with effector memory phenotypes are more abundant in the endometrium compared to the endocervix [104]. There are increased numbers of NK cells in the uterus compared to blood and these cells vary cyclically with the menstrual cycle [102]. Importantly, endometrial cells express the HIV co-receptors CCR5 and CXCR4 [105]. The uterine mucosa is enriched with lymphatic tissue which responds to hormonal stimulation (predominantly progesterone): lymphoid aggregates develop during the proliferative phase of the menstrual cycle and are largest in the secretory phase [106]. Recent data suggests that the latent HIV reservoir is at least partially maintained by clonal expansion of integrated HIV DNA [107,108]. Although not yet studied, it is plausible that this mechanism may contribute an additional source of latently infected cells in particular in the upper female genital tract. Lastly, sex hormone responsive tissue such as the endometrial tissue is subject to epigenetic regulation, and chromatin-remodelling mediators such as histone deacetylators are variably expressed at different phases of the menstrual cycle [109]. Less is known about the immune environment of the fallopian tube, but in addition to its role in fertilization, the tubes serve as a passageway for pathogens entering the pelvis and have a heterogeneous population of cells important for both innate and adaptive immunity [110]. As in the uterus, CD8^+^ T cells are more prevalent than CD4^+^ T cells with a predominant population of central memory T cells [110]. With regard to the ovary, there is very little or no information connecting that tissue with cells that could potentially harbour replication-competent HIV, or with the production of factors that have an impact on reservoir maintenance. In summary, although tantalizing evidence exists suggesting an important role for the female genital tract in the maintenance of the HIV reservoir and in the development of HIV specific immune response, the role that these compartments may play in reservoir dynamics is largely unknown.

### Sex differences in pharmacokinetics

Sex-based differences in the pharmacokinetics, bioavailability, distribution, and metabolism of drugs have been reviewed [32] and differences in the clearance of ART have been described [111]. Factors such as body weight, fat distribution, intestinal absorption, protein binding and affinity, metabolism and excretion, and other metabolic functions differ between sexes [112] and might affect the distribution of the HIV reservoir within anatomic compartments. Drug interactions between ART, contraceptive agents, and other medications are an active area of research and may change the exposure of women to antiretroviral agents. Importantly, reduced antiretroviral drug tissue penetration, relative to levels in plasma or PBMC, may create conditions favourable for local maintenance and/or replenishment of the HIV reservoir during peripherally suppressive ART (so-called pharmacological sanctuaries) [113–116].

Antiretroviral drug delivery to tissues is not uniformly distributed. In general, the concentrations of non-nucleoside reverse transcriptase inhibitors (NNRTIs) and protease inhibitors are lower in the female genital tract, relative to plasma, whereas the concentrations of NRTIs, such as emtricitabine or zidovudine, are greater [113,116]. There are exceptions to this general rule. Tenofovir and darunavir have genital tract concentrations approximately 75 and 150% higher than in blood, respectively [114,117]. Vaginal and cervical tissue concentrations of tenofovir and tenofovir diphosphate (the active intracellular metabolite) were 100-fold lower than in rectal tissue whereas emtricitabine and emtricitabine triphosphate concentrations were 10-fold higher in the vagina and cervix, when compared to rectal levels [118]. Treatment with integrase inhibitor-based therapy represents four out of the five currently recommended regimens by the US DHHS Panel [119]. Raltegravir exposure in cervicovaginal fluid is at least two-fold greater than plasma levels [120], whereas dolutegravir concentrations in female genital tract are limited. Dolutegravir exposure in cervicovaginal fluid, cervical tissue, and vaginal tissue were 6 to 10% of those observed in plasma although measured female genital tract concentrations were still greater than the protein adjusted IC90 in >90% of samples [121].

In most studies, drug concentrations in cervicovaginal lavage fluid are used as proxies for tissue concentrations and even less is known about the upper female reproductive tract. The cyclical sloughing and regeneration of the endometrium with its rich population of immune cells adds a unique and understudied variable to the reservoir dynamics in this tissue compartment. Defining the relationship between penetration of ART and virus evolution in the upper and lower female genital tract (as well as other compartments) is an active area of investigation.

### HIV latency and transcriptional activity in women

Sex differences in HIV reservoir dynamics are an underexplored area of research. One recent multicentre study investigated predictors of high HIV DNA levels in 522 HIV-positive individuals who were receiving ART for more than three years and had undetectable HIV RNA levels for more than two years. Interestingly, women were more likely to achieve a low level of HIV DNA (defined as <150 copies/10^6^ peripheral cells) in multivariate analysis [122].

*In vitro* data suggest possible effects of sex on measures of interest in eradicating HIV, including infection of and replication in target cells, cellular transcription, and HIV promoter activity [123–131]. Oestrogen receptors can bind to Sp-1, AP-1, and NF-κB subunits, transcription factors with binding sites in the HIV long terminal repeat (LTR) [132]. Estradiol has been reported to inhibit HIV replication in PBMCs at the level of transcription through oestrogen receptor α- and β-catenin-dependent mechanisms [129,133]. A recent unpublished study provided evidence that estradiol may inhibit HIV transcriptional reactivation in a sex-specific manner [134]. *Ex vivo* experiments that used PBMC isolated from treated, virologically suppressed HIV-positive individuals (males and females) demonstrated that estradiol at physiologic concentrations blocked T cell receptor-stimulated increases in partially spliced intracellular HIV *env* mRNA transcription in cells collected from women, but less so in men. Similarly, blocking oestrogen receptors (ESR)-1 and its upstream modulator steroid receptor co-activator (SRC)-3 did enhance reactivation of latent HIV provirus in primary T cells, whereas ESR-1 agonists inhibited HIV transcription. These data suggest that estradiol may be a potent inhibitor of viral reactivation and that important differences might exist between men and women for virus transcriptional reactivation. Although these data are extremely intriguing, the effects of estradiol (and other sex hormones, such as progestins) *in vivo* on HIV cure strategies will require a rigorous evaluation.

### Considerations for HIV cure research in women

Several barriers to participation in clinical trials exist for women; the inclusion of women in randomized clinical trials has historically been low [135]. Significant efforts to address this issue have been made and legislation in the United States has led to the creation of several offices within governmental agencies tasked with overseeing this mandate. Significant successes have been documented [136], although important gaps in specific areas remain. For example, a recent review of peer-reviewed publications in the cancer field found that the enrolment of women in treatment trials during the years 2001–2010 (40.2%) remained very close to what it had been in 1990–2000 (38.6%), despite federal programs aimed at increasing the enrolment of women [137]. Similarly, a review in the field of cardiovascular disease found that women remain underrepresented in National Institutes of Health (NIH)-supported cardiovascular randomized control trials [138]. A recent review summarized participation of females in global HIV cure research: Of 12,946 participants in 125 studies and 119 publications, only 2323 (17.9%) were women, with a percentage ranging from 0 to 89% per study (median 11%), and 32 studies (out of 125, 26%) reported no women enrolled [6]. The authors found a trend for studies reporting no women (or not reporting number of women) to have taken place in the United States. Of the 2323 women included in these studies, less than 1% participated in cell therapy or reactivation studies, whereas more than half of all female participants took part in studies of immune modulation. Although the overall percentage of women enrolled in global cure trials (17.9%) is similar to the sex distribution of the HIV-positive population in Western and Central Europe, North America, and Australia (where most cure research is conducted) this study found that more than one-fourth of the studies enrolled no women. Another recent review found similar results with a median of 11% of women enrolment in HIV cure studies and no significant increase over time [139]. In comparison, just over half of all NIH-funded clinical research participants (across all institutes) are women [140], but this number is enriched by several large single sex studies [141]. The lack of enrolment of women in certain areas is critically important because it prevents study findings from being truly generalizable. If sex-specific questions are not asked (or are unable to be answered because of lack of enrolment), this may have important future clinical implications.

The FDA considers treated, virologically suppressed, HIV-positive individuals in the same light as healthy subjects and the safety considerations for this group are greater than for individuals with underlying illnesses. Trials involving cure strategies may not offer direct benefit to the participants, often involve agents that are toxic, and early studies may not contain clinically relevant endpoints. The experience with the teratogenic effects of thalidomide led to the exclusion of women from clinical trials in 1977, a decision that was reversed in 1993 [136]. Although the legislative landscape has changed, attitudes may be slower to follow. Whatever the root cause, there is great reluctance to include any women of reproductive potential in early phase clinical trials because of the risks of reproductive toxicity and unintended pregnancies that could occur even when rigorous contraceptive requirements are applied.

Collection of appropriate sex-specific samples may also be a barrier to HIV cure research for women. The female pelvic exam adds time to the patient encounter and may be a deterrent to both participant and provider. Samples need to be collected during the different phases of the menstrual cycle to account for different hormonal environments in women, and collections at multiple time points may provide an additional barrier. There is a growing interest in developing methods for self-collection using swabs and devices such as menstrual cups. Many types of self-collected samples have been identified as equivalent to provider-collected samples [142,143], and methods are being developed that will ensure comparability of specimen collection across different sites. Barriers to self-collection exist, however, and some international and domestic community members report that cultural and religious constraints and stigmas may limit sample self-collection [144].

Traditional recruitment practices have proven highly effective for recruiting white men into clinical trials in the United States, Europe, and Australia. Competing priorities for women, which include childcare, home responsibilities, and shift work, often make these strategies less successful. Possible reasons for women's lack of participation might be that clinical trials involving HIV cure are often complex and require significant commitment from the participants. Study visits are long and frequent; there is often a requirement for tissue sampling and large volume blood collections or leukapheresis. Because of an increasingly limited financial climate and pressure to enrol studies efficiently, sites often rely on established recruitment practices and draw from the same pools of potential participants. Different strategies are needed to recruit and retain women into cure trials, yet budgetary and time constraints limit the ability of sites to engage and develop women-friendly recruitment strategies. A study of the participation of women in clinical HIV trials found that the most common reason for women's non-participation (reported by 75% of survey participants) was lack of information about research or not being offered enrolment [145]. When providing feedback on AIDS Clinical Trials Group (ACTG) trials in development, women frequently report that requiring two forms of contraception would be prohibitive to their enrolment and participation. To further address this issue, community members were surveyed at a recent international meeting and several additional barriers were identified: Aside from competing priorities (33%), women cited cultural stigma and lack of appropriate incentives as additional barriers (13%). Other common barriers were a lack of education about the clinical trials process and lack of awareness about trials currently open for enrolment (14%), and unwillingness to serve as a “guinea pig” (8%). Finally, several respondents felt that recruitment strategies are aimed at the same groups with little efforts to diversify, geographically or demographically (8%) [144]. Specific incentives that provide adequate childcare, a more nuanced approach to contraceptive requirements, and education targeted specifically at women might go a long way to addressing inequities in recruitment between the sexes.

Similarly, preclinical biomedical research is most commonly performed with male animals for a variety of reasons that include the costs and preservation of breeding stocks [146]. This bias further obscures key sex differences that could guide clinical studies. Differences between the sexes in both animal model and *in vitro* studies need to be carefully considered in experimental design and analysis. The NIH has tried to redress this issue by requiring sex balance in preclinical studies [140], but the cumulative effect of these requirements is yet to be determined and ensuring sex balance is still controversial in animal research [147,148].

## Conclusions

There are distinct sex-based biological differences that affect the natural history and immune pathogenesis of HIV infection. These factors likely affect the establishment and distribution of the HIV reservoir and need to be considered in the design of future strategies to cure HIV. Current strategies for cure require identification of the size and location of the latent HIV reservoir. Profound differences in the HIV reservoir may exist between men and women and need to be addressed. Latency reversing agents that are to be used in a “kick and kill” strategy may be partially inhibited by estradiol-mediated mechanisms and therefore less effective in women. Immunological approaches targeting HIV-infected cells might work differently in women because both cell- and antibody-based immunity contain differences between sexes. Moreover, fluctuating hormonal status represents an additional variable that needs to be carefully considered when designing curative interventions. Targeted enrolment of women in clinical trials and careful sex-based analysis will be crucial to gain further insights into sex-based differences in HIV infection and to design sex-specific approaches to HIV eradication, if required. To mitigate this disparity, we suggest the following course of action. First, sex-based analyses are imperative in order to understand the biological differences in HIV reservoir dynamics. Clinical trial protocols can be written to include sex-based comparisons so that necessary information can be collected as trials are conducted. Second, targeted enrolment of women is a necessity in all phases of clinical research. Although it may not be possible to recruit 50% women into every HIV cure study, a rate that matches population demographics must be a bare minimum. This may require changing recruitment strategies to address the unique barriers to women's participation. Finally, all publications should report the number of women enrolled, even if that number is zero. This review focused primarily on persons assigned female sex at birth and it will be crucial to expand this discussion to the transgender community.
